# Differential outcome of an antimicrobial stewardship audit and feedback program in two intensive care units: a controlled interrupted time series study

**DOI:** 10.1186/s12879-015-1223-2

**Published:** 2015-10-29

**Authors:** Linda R. Taggart, Elizabeth Leung, Matthew P. Muller, Larissa M. Matukas, Nick Daneman

**Affiliations:** Division of Infectious Diseases, Department of Medicine, St. Michael’s Hospital, 30 Bond Street, Toronto, ON M5B 1W8 Canada; Department of Medicine, University of Toronto, Toronto, Canada; Department of Pharmacy, St. Michael’s Hospital, 30 Bond Street, Toronto, ON M5B 1W8 Canada; Division of Microbiology, Department of Laboratory Medicine, St. Michael’s Hospital, 30 Bond Street, Toronto, ON M5B 1W8 Canada; Department of Laboratory Medicine and Pathobiology, University of Toronto, Toronto, Canada; Division of Infectious Diseases, Department of Medicine, Sunnybrook Health Sciences Centre, 2075 Bayview Ave, Toronto, ON M4N 3M5 Canada

**Keywords:** Drug utilization, Anti-infective agents, Bacterial infections, Drug resistance, microbial, Quality improvement, Intensive care

## Abstract

**Background:**

Antimicrobial decision making in intensive care units (ICUs) is challenging. Unnecessary antimicrobials contribute to the development of resistant pathogens, *Clostridium difficile* infection and drug related adverse events. However, inadequate antimicrobial therapy is associated with mortality in critically ill patients. Antimicrobial stewardship programs are increasingly being implemented to improve antimicrobial prescribing, but the optimal approach in the ICU setting is unknown. We assessed the impact of an audit and feedback antimicrobial stewardship intervention on antimicrobial use, antimicrobial costs, clinical outcomes and microbiologic outcomes in two ICUs with different patient populations.

**Methods:**

The audit and feedback intervention was implemented in a trauma and neurosurgery ICU (TNICU) and a medical surgical ICU (MSICU) at a 465-bed teaching hospital in Toronto, Canada. ICU patients were reviewed Monday to Friday by a physician and pharmacist with infectious diseases training. Recommendations related to appropriate antimicrobial use were presented to ICU teams during a dedicated daily meeting. A controlled interrupted time series analysis was used to compare outcomes in the 12 months before and after the intervention. Cardiovascular and coronary care ICUs served as control units.

**Results:**

Mean total monthly antimicrobial use in defined daily doses (DDD) per 1000 patient days was reduced 28 % in the TNICU (1433 vs. 1037) but increased 14 % in the MSICU (1705 vs. 1936). In the time series analysis, total monthly antimicrobial use in the TNICU decreased by 375 DDD per 1000 patient days (p < 0.0009) immediately following the intervention, followed by a non-significant downward trend in use of −9 DDD per 1000 patient days (*p* = 0.56). No significant changes in antimicrobial use were identified in the MSICU. Antimicrobial use temporarily increased in one control unit and remained unchanged in the other. There were no changes in mortality, length of stay, readmission rate, incidence of *C. difficile* infection or resistance patterns of *E. coli* and *P. aeruginosa* in either intervention unit.

**Conclusions:**

Audit and feedback antimicrobial stewardship programs can lead to significant reductions in total antimicrobial use in the ICU setting. However, this effect may be context-dependent and further work is needed to determine the ingredients necessary for success.

**Electronic supplementary material:**

The online version of this article (doi:10.1186/s12879-015-1223-2) contains supplementary material, which is available to authorized users.

## Background

Antimicrobial resistance is one of the most serious threats to public health today [1]. It is well accepted that antimicrobial use contributes to the development of antimicrobial resistance, and studies have shown that up to 50 % of antimicrobial use in clinical practice is inappropriate [2, 3]. Antimicrobial stewardship interventions are increasingly being advocated as an important strategy to increase the appropriateness of antimicrobial prescribing, with the aim of preventing or delaying the emergence of resistance [1, 3]. Potential additional benefits of more appropriate antimicrobial use include a reduction in adverse outcomes, including *Clostridium difficile* infection and drug reactions, as well as a reduction in healthcare costs [3–5]. The Centers for Disease Control and Prevention, the World Health Organization and the Infectious Diseases Society of America all endorse antimicrobial stewardship programs as an effective means to prevent the development and spread of antimicrobial resistance [1, 6, 7].

One of the most promising antimicrobial stewardship intervention strategies is prospective audit and feedback, a technique shown to reduce antimicrobial use in randomized-controlled trials [8, 9]. While most studies evaluating prospective audit and feedback programs have been conducted on medical and surgical wards, intensive care units (ICUs) may be the setting with the greatest potential impact [8–10]. The majority of critically ill patients receive antimicrobials and as a result, these units often have high levels of antimicrobial resistance [10, 11]. On the other hand, inadequate initial therapy has been associated with mortality in critically ill patients [12]. To date, there have been few well-conducted studies evaluating the impact of audit and feedback in ICUs [11, 13–16]. We recently introduced an audit and feedback program into two ICUs at St. Michael’s Hospital. We used interrupted time series analysis to evaluate the impact of our audit and feedback program on antimicrobial use in each of the two ICUs separately.

## Methods

### Study Design

This study evaluated changes in antimicrobial use associated with implementation of an antimicrobial stewardship audit and feedback program using a controlled interrupted time series design [17]. We hypothesized that implementation of audit and feedback would lead to reduced antimicrobial use in both units.

### Study Setting and Population

This study was performed in four adult ICUs at St. Michael’s Hospital, a 465-bed academic teaching hospital in Toronto, Ontario, Canada. The intervention ICUs included a 19-bed trauma and neurosurgery ICU (TNICU) and a 24-bed medical and surgical ICU (MSICU). The control ICUs included a 15-bed cardiovascular surgery ICU (CVICU) and a 10-bed cardiac ICU (CICU).

Antimicrobial use and other outcomes (see below) were collected for all patients admitted to the ICUs during the study period. Approval was obtained from the Research Ethics Board at St. Michael’s Hospital. The Research Ethics Board waived the need for informed consent since the study used anonymous, aggregate, retrospective data.

### Intervention

The audit and feedback intervention was introduced in the TNICU on April 1, 2013 and in the MSICU on April 15, 2013. The pre-intervention and post-intervention periods were defined as April 1, 2012 to March 31, 2013 (pre-intervention) and May 1, 2013 to April 30, 2014 (post-intervention).

During the pre-intervention period, antibiotic selection was performed at the discretion of the respective ICU teams. During the post-intervention period, an infectious diseases trained pharmacist and physician reviewed all patients admitted to the intervention ICUs daily (weekdays only). Patients who remained in the ICU were reassessed every weekday until ICU discharge. Prescribed antimicrobials, as well as microbiology, laboratory and diagnostic imaging results were reviewed. During a daily, dedicated 30 minute meeting, the ICU team presented additional clinical details for each patient and the stewardship team provided recommendations on antimicrobial use to the team. Recommendations were made verbally and documented in the chart only if requested by the ICU team. The ICU team maintained prescribing autonomy. For patients followed by the infectious diseases service, recommendations were provided to the infectious diseases team, rather than the ICU team, to avoid conflicting advice. Advice was not provided on patients with cystic fibrosis (CF) as their antibiotic management was determined by a separate CF service, whose physicians have greater expertise in the management of this patient population.

This initiative was part of an Ontario-wide quality improvement project (Council of Academic Hospitals of Ontario Antimicrobial Stewardship Program in Intensive Care Units Project) to introduce audit and feedback programs into ICUs.

### Outcomes

The primary outcome was total systemic (oral or parenteral) antimicrobial use in each ICU, measured in defined daily doses (DDD) per 1000 patient days per month [http://www.whocc.no/atc_ddd_index/]. Antimicrobial data was acquired from the pharmacy department as total grams dispensed to the unit per month (see Additional file 1). Patient days were obtained from the hospital’s administrative database.

Secondary outcome measures included the use of pre-specified antibiotic agents or classes, antimicrobial costs, antimicrobial susceptibility for *Escherichiacoli* and *Pseudomonasaeruginosa*, *Clostridiumdifficile* infection incidence, and clinical outcomes, including monthly ICU mortality rates, ICU length of stay and 48 hour ICU readmission rates.

Antimicrobial costs were calculated as Canadian dollars per patient day per month and were obtained from the pharmacy database. The number and antimicrobial susceptibility of *P. aeruginosa* and *E. coli* isolates from clinical samples were assessed. These organisms were selected a priori since they were the two most commonly isolated Gram negative organisms in our intervention ICUs. Only the first isolate per patient per hospital stay was included, unless there was a change in antimicrobial susceptibility. In this case, subsequent isolates with additional antimicrobial resistance were also included. Specimens were accepted from all clinical sites cultured with two exceptions. Respiratory specimens from patients with cystic fibrosis were excluded since these patients are often chronically colonized with multi-drug resistant organisms that, in most instances, reflect antimicrobial use prior to arrival in the ICU. Additionally, screening swabs collected for infection control purposes were not included. Susceptibility data was obtained from the clinical microbiology laboratory information system.

Incidence rates of nosocomial *C. difficile* infection were calculated based on prospective surveillance conducted by Infection Prevention and Control. Clinical outcomes, including ICU mortality rates, ICU length of stay and 48 hour ICU readmission rates, were available via the Critical Care Information System (CCIS) [http://www.health.gov.on.ca/en/pro/programs/criticalcare/ccis.aspx].

In addition to the above outcomes, age, sex, admitting diagnosis, ventilator utilization ratio (calculated as ventilator days divided by patient days) and mean multiple organ dysfunction score were obtained using data from the CCIS. Other factors likely to influence antimicrobial use, including monthly rates of febrile respiratory illness and influenza, were also collected. Finally, data related to cystic fibrosis was documented. St. Michael’s Hospital has the largest adult CF program in North America [http://www.stmichaelshospital.com/programs/cysticfibrosis/index.php]. Patients with CF frequently receive prolonged durations of multiple, broad spectrum antimicrobials at high doses. Therefore, data collection included the number of patient days per month in each unit attributable to patients with cystic fibrosis (through International Classification of Diseases 10^th^ version - ICD-10 codes) as this was a potential confounder with respect to overall antimicrobial use.

### Controls

The CVICU and CICU served as control ICUs because these units did not receive the intervention. There was minimal overlap between attending physicians in control and intervention units. H2 blocker and proton pump inhibitors, measured in DDD per 1000 patient days, were used as negative tracer medications, since prescription of these agents should not have been affected by the intervention.

### Statistical Analysis

The primary outcome was assessed by segmented regression analysis of interrupted time series data [17]. This method estimates changes in the level and trend for the outcome (i.e. antimicrobial use) after the intervention while controlling for pre-existing trends and temporal confounders. The analysis was performed separately for each of the intervention and control ICUs as well as for each of the tracer medications.

Traditional sample size calculations are not appropriate for time series analysis. Instead, it is recommended that there are a minimum of 12 data points before the intervention and 12 data points afterwards as in our study [17]. Autocorrelation was assessed by computing the Durbin-Watson statistic. Since evidence of autocorrelation was detected, all analyses were performed using autoregression in SAS (Version 9.4, Cary, North Carolina) with correction for first and second order autocorrelation using the maximum likelihood method. The assumptions of normality, homoscedasticity, and linearity were assessed using the Q-Q plot of residuals, plot of residuals against predicted values and plots of residuals against each variable in the regression model respectively. This same method was used to assess changes in tracer medications.

Categorical variables were assessed using the Chi-square test or Fisher’s exact test, continuous variables were assessed using the t-test or Wilcoxon rank sum test, and rates were assessed using incidence rate ratios.

All tests of significance were two-tailed and a *p*-value less than 0.05 was considered statistically significant. For the analyses of specific antibiotic agents and classes and for the analyses of resistance of organisms, Bonferroni corrections were used to correct for multiple hypothesis testing; for the classes of antimicrobials and individual antibiotics, a *p*-value of < 0.0028 was considered statistically significant, and for resistance tests, a *p*-value of < 0.0033 was considered significant. Statistical analysis was performed using SAS (Version 9.4, Cary, North Carolina) with the exception of incidence rate ratios, where Stata (Version 13, College Station, Texas) was used.

## Results

### Patient Characteristics

During the pre-intervention period, 1330 patients were admitted to the TNICU, corresponding to 6049 patient days, and 1305 patients were admitted to the MSICU, corresponding to 7230 patient days. In the post-intervention period, there were 1387 patients admitted to the TNICU, making up 6254 patient days, and 1369 patients admitted to the MSICU, for a total of 7488 patient days. There were no significant differences in sex, rates of febrile respiratory illness or influenza between the two intervention periods (Table 1). In the TNICU, there were minor differences in admitting diagnosis between the two periods. In the MSICU, there were differences in age and admitting diagnosis between the two periods. The mean multiple organ dysfunction score in the MSICU was lower in the post-intervention period. The most significant difference was a four-fold increase in patient days attributable to patients with cystic fibrosis in the post-intervention period in the MSICU (*p* < 0.0001).

**Table 1:** Patient characteristics for those in the trauma and neurosurgery intensive care unit and medical surgical intensive care unit during the pre- and post-intervention periods.

Unit	Characteristic	Pre-intervention period	Post-intervention period	*p*-value
TNICU				
	Admissions	*n* = 1330	*n* = 1387	
	Age, mean (SD)	55 (18) ^b^	56 (18)	0.12
	Male	757 (57)	801 (58)	0.66
	Admitting diagnosis			0.0086
	Neurological	879 (66)	937 (68)	
	Trauma	327 (25)	364 (26)	
	Other	124 (9)	86 (6)	
	Ventilator utilization ratio	0.59	0.60	0.80
	Multiple organ dysfunction score, mean (SD)	2.39 (0.34)	2.32 (0.24)	0.56^*a*^
	Febrile respiratory illness rate, cases per 1000 patient days	0.5	0.3	0.66
	Influenza rate, cases per 1000 patient days	0	0	1.00
	Patient days attributable to cystic fibrosis	0 (0)	0 (0)	–
MSICU				
	Admissions	*n* = 1305	*n* = 1369	
	Age, mean (SD)	61 (17)	59 (18)	0.016
	Male	784 (60)	833 (61)	0.68
	Admitting diagnosis			0.0082
	Respiratory	192 (15)	265 (19)	
	Gastrointestinal	142 (11)	123 (9)	
	Neurological	88 (7)	95 (7)	
	Other	883 (68)	886 (65)	
	Ventilator utilization ratio	0.69	0.69	0.77
	Multiple organ dysfunction score, mean (SD)	4.26 (0.38)	3.83 (0.33)	0.0061^a^
	Febrile respiratory illness rate, cases per 1000 patient days	21.4	17.6	0.099
	Influenza rate, cases per 1000 patient days	1.7	2.1	0.52
	Patient days attributable to cystic fibrosis	68 (1)	277 (4)	<0.0001

Data are number (%) unless otherwise indicated. All p-values calculated using Chi-square unless otherwise noted. SD, standard deviation; TNICU, trauma and neurosurgery intensive care unit; MSICU, medical surgical intensive care unit. ^*a*^t-test; ^b^2 data points missing

### Antimicrobial Use

#### TNICU

**Fig. 1:**
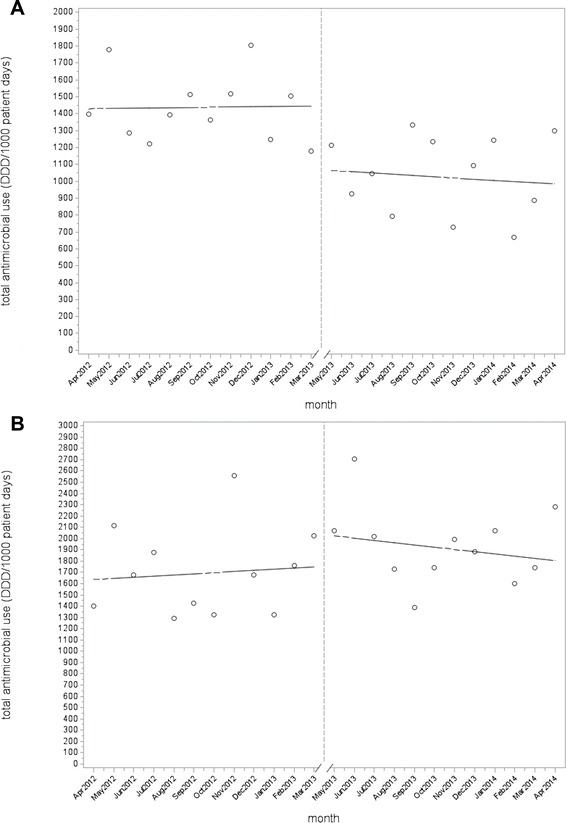
Total antimicrobial use per month pre- and post-intervention for the trauma and neurosurgery intensive care unit (**a**) and the medical surgical intensive care unit (**b**). The time series analysis demonstrated a significant decrease in the level of antimicrobial use in the trauma and neurosurgery intensive care unit by 375 defined daily doses per 1000 patient days immediately after the intervention (*p* = 0.0009) but no significant change in antimicrobial use in the medical surgical intensive care unit.

The mean total monthly antimicrobial use in the TNICU decreased by 28 % from 1433 DDD per 1000 patient days to 1037 DDD per 1000 patient days after the intervention. Time series analysis demonstrated a significant decrease in the level of antimicrobial use by 375 DDD per 1000 patient days immediately after the intervention (standard error, 94; *p* = 0.0009) (Table 2, Fig. 1). There was no significant change in the trend of antimicrobial use. With respect to specific agents and classes of antimicrobials, there was a significant reduction in antibacterials by 29 % (*p* = 0.0001), antibiotics with activity against *Pseudomonas* species by 44 % (*p* < 0.0001) and fluoroquinolones by 80 % (*p* < 0.0001) (Table 3) .

**Table 2:** Autoregressive model for total antimicrobial use per month measured in defined daily doses per 1000 patient days for intervention and control intensive care units.

Unit	Baseline level	Baseline trend	Change in level (standard error)	*p*-value	Change in trend (standard error)	*p*-value
TNICU	1427	1	−375 (94)	0.0009	−9 (15)	0.56
MSICU	1626	10	297(249)	0.25	−30 (35)	0.40
CVICU	1009	−3	169(177)	0.35	−12 (25)	0.63
CICU	591	−7	454 (128)	0.0023	−50 (19)	0.017

TNICU, trauma and neurosurgery intensive care unit; MSICU, medical surgical intensive care unit; CVICU, cardiovascular intensive care unit; CICU, cardiac intensive care unit.

**Table 3:** Comparison of the use of specific antimicrobial classes and agents measured in defined daily doses per 1000 patient days in the pre- and post-intervention periods. P-values meeting the pre-specified Bonferroni corrected significance threshold are bolded.

Unit	Class or Agent	Pre-intervention mean (SD)	Post-intervention mean (SD)	*p*-value
TNICU				
	antibacterials	1409 (203)	1001 (232)	**0.0001**
	antibacterials with antipseudomonal activity	349 (89)	195 (62)	**<0.0001**
	antifungals	24(24)	36 (25)	0.33
	penicillin	15 (21)	19 (24)	0.63
	ampicillin	86 (67)	52 (39)	0.24
	cloxacillin	337(127)	235(128)	0.068
	piperacillin-tazobactam	72 (15)	71 (30)	0.71
	cefazolin	189 (55)	138 (43)	0.028
	ceftriaxone	78 (35)	67 (41)	0.29
	ceftazidime	52 (40)	34 (21)	0.41
	ertapenem	4 (7)	3 (7)	0.47
	meropenem	11 (18)	22 (32)	0.23
	imipenem	9 (10)	16 (14)	0.20
	fluoroquinolones	210 (81)	43 (32)	**<0.0001**
	TMP-SMX	76 (73)	83 (57)	0.67
	azithromycin	12 (13)	21 (16)	0.20
	vancomycin	203 (110)	134 (52)	0.068
	aminoglycosides	4 (8)	8 (10)	0.18
MSICU				
	antibacterials	1547 (274)	1715 (263)	0.16
	antibacterials with antipseudomonal activity	445 (136)	588 (196)	0.028
	antifungals	177 (195)	202 (124)	0.14
	penicillin	55 (48)	44 (68)	0.29
	ampicillin	81 (58)	94 (89)	0.98
	cloxacillin	166 (119)	136(66)	0.93
	piperacillin-tazobactam	197 (53)	200 (34)	0.98
	cefazolin	68 (29)	83 (30)	0.35
	ceftriaxone	97 (27)	113 (40)	0.48
	ceftazidime	28 (21)	32 (29)	0.98
	ertapenem	19 (24)	15 (15)	0.77
	meropenem	28 (48)	55 (51)	0.033
	imipenem	49 (35)	47 (28)	0.84
	fluoroquinolones	138 (49)	180 (60)	0.078
	TMP-SMX	118 (93)	85 (41)	0.63
	azithromycin	101 (42)	132 (53)	0.11
	vancomycin	153 (38)	175 (43)	0.18
	aminoglycosides	29 (34)	55 (43)	0.088

All *p*-values calculated using Wilcoxon rank sum test. SD, standard deviation; TNICU, trauma and neurosurgery intensive care unit; MSICU, medical surgical intensive care unit; TMP-SMX, trimethoprim-sulfamethoxazole.

#### MSICU

The mean total monthly antimicrobial use in the MSICU was 1705 DDD per 1000 patient days before the intervention and 1936 DDD per 1000 patient days after the intervention. The time series analysis showed a non-significant increase in level of antimicrobial use of 297 DDD per 1000 patient days (standard error, 249; *p* = 0.25) and a non-significant decreasing trend in antimicrobial use of −30 DDD per 1000 patient days per month (standard error, 35; *p* = 0.40) after the intervention (Table 2, Fig. 1). There were no significant changes in the use of the specific agents or classes of antimicrobials (Table 3).

### Control ICUs

The mean total monthly antimicrobial use in the CVICU was 969 DDD per 1000 patient days before the intervention and 1071 DDD per 1000 patient days after the intervention. The time series analysis did not show any significant change in level or trend of antimicrobial use after the intervention. The mean total monthly antimicrobial use in the CICU was 545 DDD per 1000 patient days before the intervention and 599 DDD per 1000 patient days after the intervention. Time series analysis demonstrated a significant increase in the level of antimicrobial use by 454 DDD per 1000 patient days (standard error, 128; *p* = 0.0023) immediately coinciding with the onset of the intervention period, but a significant decrease in the trend of antimicrobial use of −50 DDD per 1000 patient days per month thereafter (standard error, 19; *p* = 0.017) (Table 2).

### Tracer Medications

There were no significant changes in the level or trend of H2 blocker or proton pump inhibitor use in the TNICU or MSICU post-intervention (Table 4).

**Table 4:** Autoregressive model for use of tracer medications per month in each intervention intensive care unit measured in defined daily doses per 1000 patient days.

Unit	Variable	Baseline level	Baseline trend	Change in level (standard error)	*p*-value	Change in trend (standard error)	*p*-value
TNICU							
	H2 blockers	611	−8	48 (107)	0.66	10 (15)	0.52
	Proton pump inhibitors	194	10	−113 (114)	0.33	−2 (17)	0.91
MSICU							
	H2 blockers	329	1	−82 (53)	0.14	3 (8)	0.75
	Proton pump inhibitors	924	−8	301 (151)	0.061	4 (21)	0.86

TNICU, trauma and neurosurgery intensive care unit; MSICU, medical surgical intensive care unit.

### Antimicrobial Costs

The mean total cost of antimicrobials in the TNICU decreased from $18.40 per patient day (standard deviation $4.03 per patient day) before the intervention to $14.53 per patient day (standard deviation $4.48 per patient day) after the intervention (*p* = 0.017). There was no significant change in the mean cost of antimicrobials in the MSICU with a mean total cost of antimicrobials of $33.87 per patient day (standard deviation $19.42 per patient day) before the intervention and $40.29 (standard deviation $15.88 per patient day) after the intervention (*p* = 0.14).

### Clinical Outcomes

There were no significant changes in the TNICU or MSICU mortality, length of stay in the ICU or proportion of patients readmitted to the ICU between the pre- and post- intervention periods (Table 5).

**Table 5:** Comparison of clinical outcomes in each intervention unit in the pre- and post-intervention periods.

Unit	Variable	Pre-intervention	Post-intervention	*p*-value
TNICU				
	Discharges	*n* = 1302	*n* = 1358	
	ICU mortality	86 (7)	115 (8)	0.069
	ICU length of stay in days, mean (SD)	4.7 (0.6)	4.6 (0.6)	0.38 ^*a*^
	Readmission to unit within 48 hours	17 (2)	19 (2)	0.81
MSICU				
	Discharges	*n* = 1247	*n* = 1307	
	ICU mortality	140 (11)	147 (11)	0.99
	ICU length of stay in days, mean (SD)	5.5 (0.8)	5.4 (1.0)	0.76 ^*a*^
	Readmission to unit within 48 hours	28 (3)	33 (3)	0.62

Data are number (%) unless otherwise indicated. All *p*-values calculated using Chi-square unless otherwise noted. SD = standard deviation; TNICU, trauma and neurosurgery intensive care unit; MSICU, medical surgical intensive care unit.

^*a*^Wilcoxon rank sum test

### Microbiologic Outcomes

There were no statistically significant differences in the antimicrobial susceptibility of *E. coli* or *P. aeruginosa* isolates in the TNICU or the MSICU between the pre- and post-intervention periods at the pre-specified Bonferroni corrected significance threshold of 0.0033 (Table 6). The rate of *C. difficile* infection in the TNICU decreased from 0.66 cases per 1000 patient days pre-intervention to 0.48 cases per 1000 patient days post-intervention, however, the result was not statistically significant (*p* = 0.69). There was a non-significant decrease in the rate of *C. difficile* infection in the MSICU from 1.5 cases per 1000 patient days pre-intervention to 0.80 cases per 1000 patient days post-intervention (*p* = 0.21). A post-hoc analysis revealed there was also a non-significant decrease in the incidence of *C. difficile* infection in both control ICUs.

**Table 6:** Susceptibility of *E. coli* and *P. aeruginosa* isolates to commonly used antibiotics.

Unit	Organism and antibiotic	Pre-intervention	Post-intervention	*p*-value
TNICU				
	*E. coli*			
	ampicillin	42/83 (51)	40/76 (53)	0.80
	cefazolin	70/83 (84)	61/76 (80)	0.50
	cefotaxime	72/83 (87)	64/76 (84)	0.65
	ciprofloxacin	68/83 (82)	60/76 (79)	0.64
	TMP-SMX	66/83 (80)	61/76 (80)	0.91
	piperacillin-tazobactam	70/83 (84)	63/76 (83)	0.81
	imipenem	85/85 (100)	76/77 (99)	0.48^*a*^
	gentamicin	77/83 (93)	70/76 (92)	0.87
	tobramycin	76/83 (92)	70/76 (92)	0.90
	*P. aeruginosa*			
	ceftazidime	27/30 (90)	22/23 (96)	0.62^*a*^
	ciprofloxacin	27/30 (90)	18/23 (78)	0.27^*a*^
	piperacillin-tazobactam	27/30 (90)	22/23 (96)	0.62^*a*^
	imipenem	30/30 (100)	20/23 (87)	0.076^*a*^
	gentamicin	28/30 (93)	23/23 (100)	0.50^*a*^
	tobramycin	30/30 (100)	23/23 (100)	-
MSICU				
	*E. coli*			
	ampicillin	37/95 (39)	30/81 (37)	0.79
	cefazolin	65/95 (68)	59/81 (73)	0.52
	cefotaxime	73/95 (77)	64/81 (79)	0.73
	ciprofloxacin	46/95 (48)	49/81 (60)	0.11
	TMP-SMX	58/95 (61)	43/81 (53)	0.29
	piperacillin-tazobactam	64/95 (67)	59/80 (74)	0.36
	imipenem	93/95 (98)	81/82 (99)	1.00^*a*^
	gentamicin	76/95 (80)	73/81 (90)	0.063
	tobramycin	72/95 (76)	72/81 (89)	0.025
	*P. aeruginosa*			
	ceftazidime	50/64 (78)	42/58 (72)	0.46
	ciprofloxacin	58/64 (91)	41/58 (71)	0.0049
	piperacillin-tazobactam	50/64 (78)	40/58 (69)	0.25
	imipenem	51/64 (80)	41/58 (71)	0.25
	gentamicin	58/64 (91)	48/58 (83)	0.20
	tobramycin	62/64 (97)	56/58 (97)	1.00^*a*^

Data are number of isolates susceptible/total number of isolates tested (%). All p-values calculated using Chi-square unless otherwise noted. A Bonferroni corrected significance threshold of 0.0033 was used. TMP-SMX, trimethoprim-sulfamethoxazole.

^*a*^Fisher’s Exact test

## Discussion

In this study, we demonstrated that an audit and feedback antimicrobial stewardship intervention, when introduced simultaneously into two distinct ICUs, yielded different results. In the TNICU, an immediate and clinically significant drop in antimicrobial use was observed with an overall reduction of 28 %. In the MSICU, there was no appreciable change in antimicrobial use attributable to the intervention. No significant immediate reductions were noted in the control ICUs or with the tracer medications, suggesting that the intervention was responsible for the change in the TNICU. However, this does not explain why the intervention was successful in only one of two intervention ICUs, an unexpected finding given that the stewardship team and the format of the intervention were identical in both units.

The magnitude of the result in the TNICU is similar to results reported in a systematic review of stewardship interventions in critical care units, where reductions in antimicrobial use of 11–38 % were observed [13]. Studies using audit and feedback strategies in critical care units showed reductions in antimicrobial use ranging from 8 %–22 % [11, 15, 16, 18, 19]. However, some stewardship studies have focused their intervention and outcome on ‘targeted antimicrobials’ [20]. As a result, these studies have shown significant reductions in targeted antimicrobials, without measuring compensatory increases that can occur in other antimicrobials, a phenomenon known as “squeezing of the balloon” [21]. Therefore, a strength of our study, is that it demonstrated a reduction in overall antimicrobial use, rather than simply the use of specific agents.

Another important finding in the TNICU was a 44 % reduction in the use of antimicrobials effective against *Pseudomonas* species. This is important, since *Pseudomonas* species are intrinsically drug resistant organisms and thus it is likely beneficial to conserve antibiotics used to treat these organisms. Furthermore, the reduction in fluoroquinolone use was important since these antibiotics have been associated with a low threshold for emergence of resistance as well as an increased risk of development of *Clostridium difficile* infection [22, 23]. Furthermore, substantial overall cost savings of 21 % were seen in the post-intervention period.

In the MSICU, antimicrobial use was unchanged. There are several potential factors that may have contributed to the absence of a measurable effect of the stewardship intervention. First, because appropriateness of therapy was not measured as one of our study outcomes, it is possible that antimicrobial prescribing was already ‘more appropriate’ in the MSICU. Alternatively, because the MSICU patient population is older, more critically ill and more likely to have an infection present at the time of admission, differences in patient population may have contributed to these findings. Overall antibiotic use may have been more driven by initial empiric therapy, potentially resulting in the ICU team being less likely to follow stewardship recommendations. Finally, the two units have different leadership, cultures, educational structures and decision-making processes.

One additional consideration is that in the post-intervention period there was a 4-fold increase in the number of patient days associated with patients with cystic fibrosis in the MSICU. CF patients often harbor multi-drug resistant bacteria [24]. They are also often treated with multiple antimicrobials and higher doses of antimicrobials than other adults, thus their admission to the MSICU could significantly increase monthly antimicrobial use [25, 26]. It would have been desirable to exclude these patients from the analysis since they were not included in the intervention; however, this was not possible due to limitations related to the hospital information system used to quantify antimicrobial use, which was not able to link usage data to individual patients. Instead, the primary analysis was repeated with adjustment for the number of CF patient days per month, and the overall results did not change. There were other statistically significant differences in patient characteristics in the MSICU in the post intervention period in comparison to the pre-intervention period including a different distribution of admitting diagnoses and a lower multiple organ dysfunction score; however, these differences do not appear large enough to explain the inability to reduce antimicrobial use in this unit. There were no discernable clinically relevant changes in the types of organims isolated during the study period. Furthermore, there were no outbreaks in any of the intervention or control units throughout the study period with the exception of a vancomycin-resistant *Enterococcus* outbreak in the MSICU involving ten patients in the pre-intervention period. This represented colonization rather than clinical infection in the majority of cases and was therefore unlikely to have significantly influenced antimicrobial prescribing. Thus there were likely other factors involved in the differential outcomes between the TNICU and MSICU.

As expected, there were no significant changes in clinical outcomes including in-ICU mortality, ICU length of stay and 48 hour ICU readmission rates. There was a trend toward increased mortality in the TNICU, however, the post-intervention mortality rate was in line with TNICU annual mortality rates over the preceding 5 years, which have ranged from 6.6-8.4%. Nevertheless, with any intervention aimed at reducing inappropriate use, it is critical to ensure that there are no direct harms resulting from the intervention and we continue to monitor this metric. The lack of change in resistance patterns was not unexpected. Although stewardship interventions have been associated with protection against the emergence of resistance, a longer follow up period may be required to appreciate changes in ICU ecology [14]. This was demonstrated by Geissler et al. who found that a reduction in nosocomial infections due to antimicrobial resistant organisms was only observed 3 years after implementation of an intervention [27].

The trend toward reduction in rates of *Clostridium difficile* infection seen in both intervention ICUs seemed promising, however, rates in the control ICUs were also reduced. Therefore, the reduction was likely unrelated to the intervention. This finding highlights the importance of including controls in quasi-experimental antimicrobial stewardship research, as the incidence of a variety of outcomes, including *C. difficile,* may be due to regression to the mean, changes in local epidemiology, or non-stewardship interventions (e.g. improved hand hygiene or environmental cleaning). In our hospital, infection prevention and control interventions remained constant throughout the study period.

Our study had several limitations. First, our intervention occurred on weekdays only and thus may underestimate the potential benefit of audit and feedback. In addition, the ideal primary outcome would be appropriateness of antimicrobial therapy, rather than antimicrobial use. However, evaluating appropriateness is subjective and labor intensive, and given that the literature has consistently shown that antimicrobials are overused, a reduction in antimicrobial use, under the supervision of an infectious diseases physician and infectious diseases trained pharmacist, was a rational goal [28]. Furthermore, the use of DDDs to quantify antimicrobial use can be problematic, since critically ill patients may routinely receive higher daily doses for certain agents than those defined by the WHO [29]. It is possible that by converting to narrower agents, the defined daily doses will actually increase. Finally, as with many studies in the field of antimicrobial stewardship, temporal confounding is a concern. However, our controlled, interrupted time series design is more robust than the before and after analyses or uncontrolled interrupted time series designs used in most stewardship studies [20].

## Conclusions

Our results demonstrate the potential for audit and feedback to significantly reduce antimicrobial use in some, but not all, ICU settings. We also demonstrate the importance of a controlled study design in assessing the impact of stewardship on a variety of clinical outcomes, including *C. difficile* incidence. Additional research is required to understand the predictors of success for specific stewardship interventions.

## Additional file

Additional file 1:
**Antimicrobials Included in Overall Antimicrobial Use and Cost.** Antimicrobials Included in Overall Antimicrobial Use and Cost (PDF 206 kb)

## Abbreviations

CCIS: Critical Care Information System
CF: Cystic fibrosis
CICU: Cardiac intensive care unit
CVICU: Cardiovascular surgery intensive care unit
DDD: Defined daily dose
ICU: Intensive care unit
MSICU: Medical surgical intensive care unit
TNICU: Trauma and neurosurgery intensive care unit
