# Supplementary material for: Differential outcome of an antimicrobial stewardship audit and feedback program in two intensive care units: a controlled interrupted time series study
Source: BMC Infect Dis. 2015 Oct 29;15:480. doi: 10.1186/s12879-015-1223-2 (PMC4625716; doi:10.1186/s12879-015-1223-2)
Supplement: Additional file 1: — Antimicrobials Included in Overall Antimicrobial Use and Cost. Antimicrobials Included in Overall Antimicrobial Use and Cost (PDF 206 kb) [file 12879_2015_1223_MOESM1_ESM.pdf]

Antimicrobials Included in Overall Antimicrobial Use and Cost

Systemic Antibacterials
amikacin
ampicillin
amoxicillin
amoxicillin-clavulanate
atovaquone
azithromycin
aztreonam
cefaclor
cefadroxil
cefazolin
cefepime
cefixime
cefmetazole
cefotaxime
cefoxitin
cefprozil
ceftriaxone
ceftazidime
cefuroxime
cephalexin
ciprofloxacin
chloramphenicol
clarithromycin
clindamycin
cloxacillin
colistimethate sodium
daptomycin
dapsone
doripenem
doxycycline
ertapenem
erythromycin
fosfomycin
gentamicin
imipenem-cilastatin
levofloxacin
linezolid
meropenem
metronidazole
minocycline
moxifloxacin
nitrofurantoin

norfloxacin
ofloxacin
penicillin G (exception: exclude penicillin G benzathine)
penicillin V
piperacillin
piperacillin-tazobactam
quinupristin-dalfopristin
rifabutin
rifampin
sodium fusidate
sulfamethoxazole-trimethoprim
ticarcillin-clavulanate
tigecycline
trimethoprim
tobramycin
vancomycin

Systemic Antifungals
amphotericin B
anidulafungin
caspofungin
fluconazole
flucytosine
itraconazole
ketoconazole
micafungin
nystatin (oral use)
posaconazole
terbinafine
voriconazole

Systemic Antivirals
oseltamivir
zanamivir
